# Validation of a real-time quaking-induced conversion (RT-QuIC) assay protocol to detect chronic wasting disease using rectal mucosa of naturally infected, pre-clinical white-tailed deer (*Odocoileus virginianus*)

**DOI:** 10.1371/journal.pone.0303037

**Published:** 2024-06-13

**Authors:** Robert B. Piel, Susan E. Veneziano, Eric M. Nicholson, Daniel P. Walsh, Aaron D. Lomax, Tracy A. Nichols, Christopher M. Seabury, David A. Schneider

**Affiliations:** 1 U.S. Department of Agriculture, Agricultural Research Service, Animal Disease Research Unit, Pullman, Washington, United States of America; 2 Department of Veterinary Microbiology and Pathology, Washington State University, Pullman, Washington, United States of America; 3 U.S. Department of Agriculture, Agricultural Research Service, National Animal Disease Center, Virus and Prion Research Unit, Ames, Iowa, United States of America; 4 U.S. Geological Survey, Montana Cooperative Wildlife Research Unit, Missoula, Montana, United States of America; 5 Wildlife Biology Program, University of Montana, Missoula, Montana, United States of America; 6 Department of Soil Science, University of Wisconsin, Madison, Wisconsin, United States of America; 7 U.S. Department of Agriculture, Animal Plant Health Inspection Service, Veterinary Services, Fort Collins, Colorado, United States of America; 8 Department of Veterinary Pathobiology, Texas A&M University, College Station, Texas, United States of America; The University of Texas Health Science Center at Houston, UNITED STATES

## Abstract

Chronic wasting disease (CWD) is a fatal prion disease of cervids spreading across North America. More effective mitigation efforts may require expansion of the available toolkit to include new methods that provide earlier antemortem detection, higher throughput, and less expense than current immunohistochemistry (IHC) methods. The rectal mucosa near the rectoanal junction is a site of early accumulation of CWD prions and is safely sampled in living animals by pinch biopsy. A fluorescence-based, 96-well format, protein-aggregation assay—the real-time quaking-induced conversion (RT-QuIC) assay—is capable of ultra-sensitive detection of CWD prions. Notably, the recombinant protein substrate is crucial to the assay’s performance and is now commercially available. In this blinded independent study, the preclinical diagnostic performance of a standardized RT-QuIC protocol using a commercially sourced substrate (MNPROtein) and a laboratory-produced substrate was studied using mock biopsy samples of the rectal mucosa from 284 white-tailed deer (*Odocoileus virginianus)*. The samples were from a frozen archive of intact rectoanal junctions collected at depopulations of farmed herds positive for CWD in the United States. All deer were pre-clinical at the time of depopulation and infection status was established from the regulatory record, which evaluated the medial retropharyngeal lymph nodes (MRPLNs) and obex by CWD-IHC. A pre-analytic sample precipitation step was found to enhance the protocol’s detection limit. Performance metrics were influenced by the choice of RT-QuIC diagnostic cut points (minimum number of positive wells and assay time) and by deer attributes (preclinical infection stage and prion protein genotype). The peak overall diagnostic sensitivities of the protocol were similar for both substrates (MNPROtein, 76.8%; laboratory-produced, 73.2%), though each was achieved at different cut points. Preclinical infection stage and prion protein genotype at codon 96 (G = glycine, S = serine) were primary predictors of sensitivity. The diagnostic sensitivities in late preclinical infections (CWD-IHC positive MPRLNs and obex) were similar, ranging from 96% in GG96 deer to 80% in xS96 deer (x = G or S). In early preclinical infections (CWD-IHC positive MRPLNs only), the diagnostic sensitivity was 64–71% in GG96 deer but only 25% in xS96 deer. These results demonstrate that this standardized RT-QuIC protocol for rectal biopsy samples using a commercial source of substrate produced stratified diagnostic sensitivities similar to or greater than those reported for CWD-IHC but in less than 30 hours of assay time and in a 96-well format. Notably, the RT-QuIC protocol used herein represents a standardization of protocols from several previous studies. Alignment of the sensitivities across these studies suggests the diagnostic performance of the assay is robust given quality reagents, optimized diagnostic criteria, and experienced staff.

## Introduction

Chronic wasting disease (CWD) is a contagious and ultimately fatal prion disease that affects a variety of cervid species, including white-tailed deer (WTD) (*Odocoileus virginianus*) [1,2]. Disease transmission occurs through exposure to misfolded forms of the prion protein, PrP^CWD^ [3], which later accumulate in specific tissues of infected animals [4,5]. CWD exhibits a prolonged preclinical incubation period that can last several years, during which infected animals shed prions into the environment [6,7]. For these reasons, mitigating the spread of CWD is especially challenging, and effective means by which to surveil and monitor the infection status of cervid populations represent an essential management tool.

Accumulation of PrP^CWD^ is progressively observed in several lymphoid tissues associated with the alimentary tract and later in the central nervous system. PrP^CWD^ is often first observed in the medial retropharyngeal lymph nodes (MRPLNs). Later accumulation in the central nervous system is first observed in the brainstem at the level of the obex [4,5,8]. The current regulatory standard focuses on these two tissues for postmortem diagnosis using antibody-based detection methods: either immunohistochemical (IHC) staining of formalin-fixed tissues or enzyme-linked immunosorbent assay (ELISA) on tissue homogenates [9].

Early accumulation of PrP^CWD^ also occurs in other alimentary tract lymphoid tissues, including two that are accessible by biopsy in living animals, the palatine tonsils and the lymphoid follicles associated with the rectal mucosa near the rectoanal junction, the so called rectoanal mucosa-associated lymphoid tissue (RAMALT). One study tracked the lymphoid distribution of PrP^CWD^ in WTD after controlled oronasal inoculation and postmortem collection of whole tissues [10]. PrP^CWD^ was first detected, though inconsistently, in the MRPLN and palatine tonsils during the first two months. Detection in the RAMALT wasn’t observed until three and four months after inoculation. Though prion detection occurred earlier in the palatine tonsils when examined as a whole tissue, studies using biopsy samples from naturally infected WTD show little difference in the diagnostic sensitivity between palatine tonsil [11] and RAMALT [12]. As a practical matter, biopsy of the tonsil requires heavy sedation and use of a specialized biopsy instrument to retrieve comparatively limited amounts of tissue. In contrast, pinch biopsy of the rectal mucosa is achieved using forceps and scissors in restrained or sedated animals. Biopsy of the rectal mucosa can be safely repeated [13] and is utilized to screen farmed herds for CWD by IHC [9]. Reports of the diagnostic sensitivity of CWD-IHC using rectal mucosa have varied widely, ranging from 44% [14] to 91% [15]. A meta-analysis based on mock rectal mucosal biopsies from four depopulations of WTD farms yielded a pooled estimate of preclinical diagnostic sensitivity of 68% with 95% confidence limits of 49% to 82% [12]. Improved antemortem sensitivity would expand herd management strategies, especially during early infection.

The real-time quaking-induced conversion (RT-QuIC) assay is a seeded amplification assay capable of detecting minimal amounts of PrP^CWD^ in a wide variety of tissues. However, assay performance is dependent on several factors, especially the quality of the recombinant prion protein substrate. A variety of RT-QuIC protocols and laboratory-produced substrates have been applied to samples of rectal mucosa from elk [16–19] and deer [14,20,21]. Broadly, these studies demonstrate some degree of improved sensitivity with respect to CWD-IHC evaluation of rectal mucosa.

The current study determined the preclinical diagnostic performance of a standardized RT-QuIC protocol for rectal mucosa from farmed WTD, using either a commercially sourced substrate or a similarly shipped substrate produced by an experienced prion research laboratory. Performance metrics were evaluated at different cut points and considering preclinical stage of infection (early vs. late) and allelic variation in the prion protein gene (*PRNP*).

## Methods and materials

### Validation sample set

Samples were made available from a frozen archive of rectal cores that were harvested in the field at the time of depopulation. The rectal core samples used in this study come from nine farms positive for CWD in the United States and depopulated as a regulatory action. Each rectal core was removed using a boning knife, similar to the procedure shown by Keane et al. [15] and taking care to not penetrate the lumen of the rectum. Each core was individually placed in a Ziplock bag and kept frozen at -80°C. To retrieve a mock biopsy of the rectal mucosa, each rectal core was partially thawed in the laboratory on clean laboratory bench paper. A single-use razor blade was used to open the lumen of the core on the longitudinal axis. With the rectal lumen now visible, a second single-use razor blade was used to remove a mock biopsy of the rectal mucosa that purposely included the “white line”, a readily visible demarcation between rectal mucosa and anal epithelium [15]. Each mock biopsy sample was placed in an individual bag and re-frozen prior to overnight shipment to the testing laboratory where they were stored at -80°C. Fresh gloves, laboratory bench paper, and razor blades were used for each sample.

The infection status of each animal was established from the regulatory results of CWD-IHC on the medial retropharyngeal lymph nodes (MRPLNs) and obex. All infected animals were considered preclinical as clinical signs consistent with CWD were not observed prior to depopulation. Early preclinical infection was defined as PrP^CWD^ accumulation observed by CWD-IHC only in the MRPLNs whereas late preclinical infection had the additional presence of PrP^CWD^ in the obex. The number and average age of deer in each infection category, *PRNP* genotype, and sex are provided in Table 1.

**Table 1:** Contingency outcomes from RT-QuIC analyses of rectal mucosa stratified by study population characteristics.

CWD-IHC		*PRNP*			age (y)			MNPROtein				NWHC			
MRPLN and obex															
Infection Status	Stage of Infection	codon 96	codon 95	sex	*median*	*min*—*max*	*count*	*TN*	*FP*	*TP*	*FN*	*TN*	*FP*	*TP*	*FN*
ND	ND	GG	QQ	F	5		1	1				1			
				M	3.5	1.0–9.5	6	6				6			
			QH	M	4		1	1				1			
			*unk*	F	6	1.0–10.0	31^a^	30	1			31			
				M	2.8	1.0–5.0	8	8				8			
				*unk*	9		1	1				1			
		GS	QQ	F	6		1		1				1		
				M	2.5	1.0–3.5	9	9				8	1		
			QH	F	6		1	1				1			
			*unk*	F	5	1.0–9.0	10^a^	10				9	1		
				M	2.5	2.5–3.5	6	6				6			
		SS	QQ	M	3.5	2.5–4.5	3	3				3			
			*unk*	M	3.5		1	1				1			
		*unk*	*unk*	F	5	1.0–10.5	34^a^	34				34			
				M	2.5	0.4–8.0	112^b^	112				110	2		
				*unk*	2		3^c^	3				3			
POS	Early Preclinical	GG	QQ	F	4	2.0–13.0	5			1	4			1	4
				M	3	1.0–5.0	6			6				6	
			*unk*	F	6		1			1					1
				M	3	3.0–3.0	2			2				2	
		GS	QQ	F	5.5	5.0–6.0	2			1	1			1	1
				M	4.5	4.5–4.5	2				2				2
			QH	M	6		1				1				1
		SS	QQ	M	4.5	4.5–5.0	3			1	2			1	2
	Late Preclinical	GG	QQ	F	2	1.0–5.0	10			9	1			9	1
				M	3	1.0–6.0	10			10				9	1
			*unk*	F	2	1.0–5.0	3			3				2	1
				M	3	2.0–4.5	3			3				3	
		GS	QQ	F	4	4.0–4.0	2			1	1			1	1
				M	4	4.0–4.0	2			2				2	
			*unk*	M	4		1			1				1	
		*unk*	*unk*	F	8		1			1				1	
				M	4.5	0.4–8.5	2			1	1			2	
*Column Totals*:							*284*	*226*	*2*	*43*	*13*	*223*	*5*	*41*	*15*

The left most columns stratify results by study population characteristics whereas right most columns report the frequencies of contingency outcomes by source of the recombinant prion protein substrate (MNPROtein or NWHC) used in the RT-QuIC assay. Diagnostic calls based on RT-QuIC could either agree or disagree with gold standard test results (CWD-IHC on medial retropharyngeal lymph nodes and obex), resulting in the paired outcomes of true negative versus false positive (*TN* vs *FP*) and true positive versus false negative (*TP* vs *FN*). Contingency outcomes that were different between substrates are highlighted in red. The study population consisted of WTD from farms quarantined for CWD, but none were showing clinical signs of CWD at the time of depopulation.

^a, b, c^ Number of deer for which ages were not available: a = 1, b = 4, c = 2.

### Preparation of homogenate stocks of rectal mucosa

Mock biopsy samples ranged in size from 2–4 sq cm from which a 100 mg mucosal subsample was collected immediately adjacent to the white line. The subsample was homogenized in 9 volumes of 1× phosphate-buffered saline (PBS) using 0.7 mm Zirconia beads (BioSpec 11079107zx) in a bead beating grinder (Fast Prep 24 –MP bio) for 3 cycles of 45 sec at speed setting 5.5 with 5 min rest on ice between cycles. The homogenate was immediately removed from beads leaving behind any undisrupted connective tissue, aliquoted, and stored at -80°C. The stock sample homogenates were thus approximately 10% (w/v).

### Homogenate dilutions

Dilutions of stock homogenate were made with 1× PBS pH 7.4 also containing 0.1% SDS and 1× N-2 media supplement (Gibco-Fisher). The dilution nomenclature used throughout this manuscript is relative to intact tissue, such that the stock homogenate at 10% (w/v) is represented as a 10^−1^ dilution of the original sample.

### NaPTA precipitation

For precipitation by sodium-phosphotungstic acid (NaPTA), an aliquot of stock homogenate was thawed, briefly vortexed, and sonicated for 30 sec in a water bath sonicator (QSonica Q700) at amplitude 65 (~180–200 W output). The sonicated stock homogenate was then centrifuged for ~1 min in a single speed 0.2 mL benchtop centrifuge to pellet particulate material. Next, the supernatant was removed and mixed 1:100 with 1× PBS pH 7.4 containing 0.3% NaPTA and 12 mM MgCl_2_. This mixture was incubated at room temperature for 1 hour with gentle agitation. The precipitate was then pelleted by centrifugation of the mixture for 30 min at 21,000 × g. Pellets were resuspended in 1× PBS pH 7.4 containing 0.1% SDS and 1× N-2 media supplement (Gibco-Fisher). To ensure complete resuspension, alternate vortexing and sonication, as described above, were applied to pellets until fully resolubilized. For use in the blind validation study, a starting volume of 15 μL of the original 10% sample homogenate was precipitated in a total volume of 1,500 μL, and resuspended in a final volume of 150 μL, thereby representing a 10^−2^ dilution relative to the intact tissue.

### rPrP substrate

Two sources of recombinant truncated Syrian hamster prion protein (accession# K02234, residues 90–231) (Ha90 rPrP) were used as RT-QuIC substrates in this study. One was commercially produced and marketed as MNPROtein by the Minnesota Center for Prion Research and Outreach. The other was produced at the U.S. Geological Survey National Wildlife Health Center (NWHC). For production of the NWHC substrate, rPrP was expressed in DE3 *Escherichia coli* from the pET41 vector, purified from inclusion bodies using nickel immobilized metal affinity chromatography (IMAC) resin, and refolded using a guanidine hydrochloride gradient. Proteins were eluted with imidazole and subsequently dialyzed and diluted to 0.4 mg/ml in 10 mM Na_2_PO_4_ (pH 5.8). Each source shipped substrate overnight on dry ice to the testing laboratory. Substrate aliquots were stored at -80°C.

**Fig 1:**
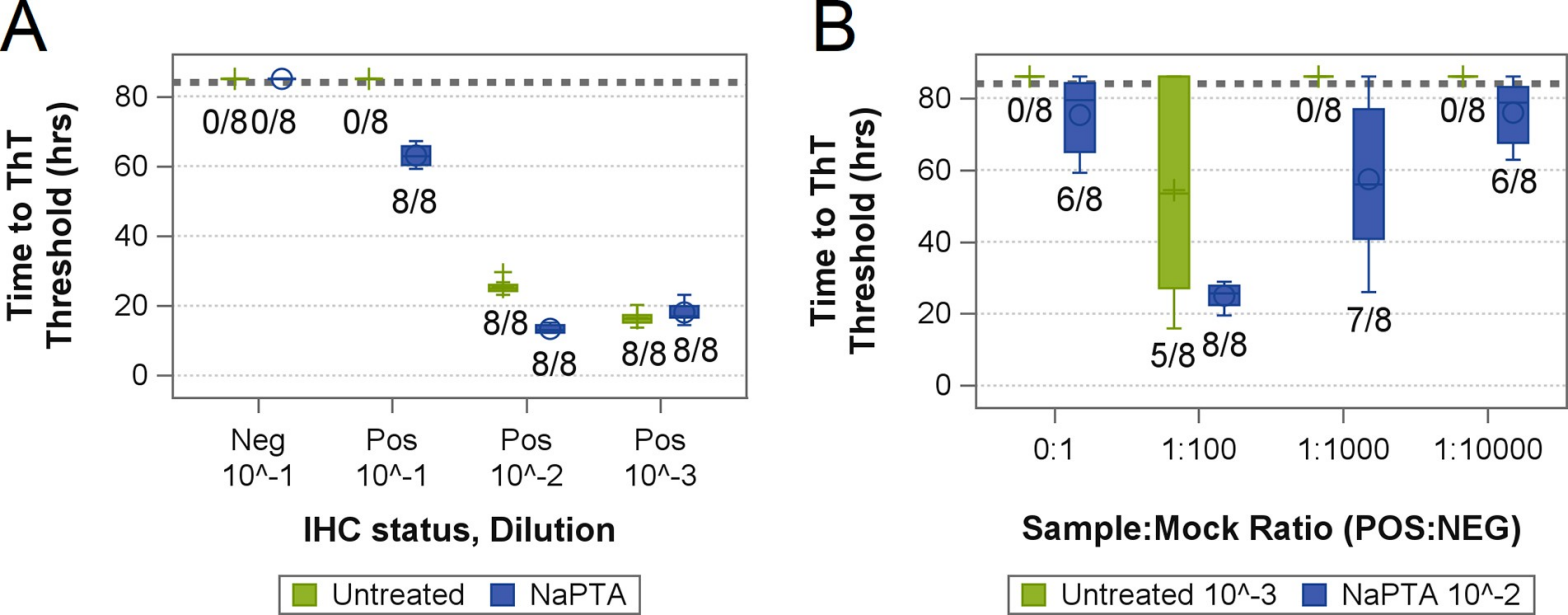
Optimization of rectal mucosa sample preparation. Boxplots showing (A) time to thioflavin T (ThT) threshold from a real-time quaking-induced conversion (RT-QuIC) assay of a rectal mucosa homogenate from a late preclinical white-tailed deer. The homogenate sample was prepared by dilution only (Untreated) or by dilution following precipitation with sodium-phosphotungstic acid (NaPTA). (B) Time to ThT threshold from an RT-QuIC assay of “mock minimal” samples. Here, the rectal mucosa homogenate from the same CWD-positive deer was spiked (at ratios from 1:100 to 1:10,000) into a rectal mucosa homogenate from a deer in which CWD was not detected. “Mock minimal” samples were prepared for RT-QuIC assay by either dilution only to 10^−3^ or by NaPTA precipitation and subsequent dilution to 10^−2^.

Diagnostic performance testing of the standardized RT-QuIC protocol revealed a difference in the time-course of RT-QuIC reactions between the substrates. Gross differences in substrate purity were not evident when subsequent production runs of each substrate were examined by denaturing gel electrophoresis and Coomassie staining (S2 Fig 1; available at Ag Data Commons repository, DOI: 10.15482/USDA.ADC/24727866).

### RT-QuIC assay

The protocol standardized for this project is a unification of the general method described by Orru et al. (2017) with additional details and modifications supported in the subsequent literature, especially those reporting sensitivity for CWD in cervid species [14,16,18,20,22–24]. The detailed standardized protocol is freely available at protocol.io (DOI: 10.17504/protocols.io.yxmvmn2y6g3p/v1) and is briefly described here.

Prepared sample homogenates (dilutions and/or resuspended NaPTA precipitations) were used as the seeding material for the RT-QuIC assay. In each reaction well, 2 uL prepared homogenate was added to 98 μL RT-QuIC assay buffer [10 mM NaPO_4_ pH 7.4, 300 mM NaCl, 1 mM EDTA, 10 μM ThT, 0.1 mg/mL rPrP]. Assays were performed in FLOUstar optima plate readers (BMG Labtech) at 42°C with shaking cycles of 1 min at 700 rpm double orbital followed by 1 min rest. Assays were performed for ~83 hours total with measurements taken at 43 min intervals. ThT fluorescence was measured with an excitation wavelength of 450 ± 10 nm and emission wavelength of 480 ± 10 nm via bottom read using 20 flashes/well. Gain was set to 1800.

### Fluorescence threshold and lag times

A standard method for assessing “positivity” in the RT-QuIC assay is to determine a critical threshold value (*ThT*_*crit*_ *ThT*_*crit*_). In the present study, a single *ThT*_*crit*_ value was calculated for each assay plate from ThT readings during the first 5 hours of the assay, a baseline period during which aggregation reactions were rarely observed. Thus, *ThT*_*crit*_ was calculated from 768 baseline period ThT values per full 96-well plate; i.e., 8 readings per well multiplied by 96 wells. *ThT*_*crit*_ was calculated as the plate *median* of ThT plus 15 times $\hat{\sigma }$ (Eq 1), where $\hat{\sigma }$ is a robust estimate of the sample standard deviation based on the median absolute deviation about the median (*MAD*; Eq 2) [25].

$${ThT}_{crit}={median}_{ThT}+15\hat{\sigma }$$ (1)

$${ThT}_{crit}={median}_{ThT}+15(MAD\times 1.4826)$$ (2)

The use of these robust measures (rather than using mean and standard deviation from the mean) was chosen to reduce the impact of outlier fluorescence values. Outlier baseline period ThT readings occasionally experienced in this study were a few authentic seeded reactions (i.e., rapidly increasing) that began between 4 and 5 hours, and initial elevated fluorescence values that decayed rapidly to a typical and stable baseline fluorescence level by 2 hours (occurring irrespective of infection status of the deer).

The time-to-threshold (hereafter, lag time) was also determined for each positive reaction as the earliest time point after the fluorescence signal exceeded *ThT*_*crit*_.

### Diagnostic performance metrics

The official diagnosis of a diseased animal was based on CWD-IHC of MRPLNs and obex tissues and served as the gold standard against which the diagnostic performance of a rectal mucosal sample analyzed by the standardized RT-QuIC protocol was measured. To facilitate a more granular investigation, deer were grouped into three stages of infection as follows. Deer with PrP^CWD^ accumulation observed by CWD-IHC in the MRPLNs but not the obex were considered early preclinical infections, those with accumulation observed in the MRPLNs and the obex were considered late preclinical infections, and deer in which PrP^CWD^ accumulation was not observed in either tissue were considered “not detected” (ND). It should be noted that CWD-IHC results were not available for the samples of rectal mucosa, which had originally been collected only as fresh frozen samples.

Diagnostic performance measures were determined using the coding templates and formulas detailed in SAS Usage Note 24170 [26]. The overall performance of RT-QuIC was measured separately for each substrate and at each ThT signal read time using a SAS non-linear mixed model procedure, PROC NLMIXED (SAS Institute Inc., Cary, NC, USA). Performance measures were estimated by fitting a saturated Poisson model that included one parameter for each cell of a 2×2 contingency table comparing the results of RT-QuIC for rectal mucosa (test assay) versus the reported CWD-IHC result for MRPLNs and obex (gold standard assay). The cells of the contingency table thus correspond to the four possible test assay comparative outcomes: True Positive (*TP*), True Negative (*TN*), False Positive (*FP*), and False Negative (*FN*). Exact 95% confidence limits for sensitivity and specificity were determined at specific times of interest for the binomial proportions from the corresponding contingency table (PROC FREQ).

The diagnostic calls for the test assay were evaluated at each of the four possible levels of replication, i.e., the criterion for a positive test result at a given timepoint could require a minimum of 1 well (RXN1), 2 wells (RXN2), 3 wells (RXN3), or all 4 wells (RXN4) to exceed *ThT*_*crit*_. The performance of these four possible RT-QuIC result criterions were compared using the area under the curve (AUC) of receiver operating characteristic (ROC) plots as determined by logistic regression (PROC LOGISTIC, ROCCONTRAST option comparing all other criterions to RXN3).

The measures of diagnostic performance included standard calculations of sensitivity (Eq 3) and specificity (Eq 4). The F1 score is the harmonic mean of precision and recall. Precision, also known as positive predictive value, is the probability that a positive test result has identified a truly positive animal (Eq 5). Recall is the probability that a truly positive animal will be recognized by the test assay, i.e., recall is just another name for sensitivity (Eq 3). The formula used to calculate the F1 score is given in Eq 6.

$$\mathrm{Sensitivity}\;(\mathrm{or}\;\mathrm{Recall})=\frac{TP}{TP+FN}$$ (3)

$$\mathrm{Specificity}=\frac{TN}{TN+FP}$$ (4)

$$\mathrm{Precision}=\frac{TP}{TP+FP}$$ (5)

$$F1\;\mathrm{score}=2\times \frac{\mathrm{precision}\times \mathrm{recall}}{\mathrm{precision}+\mathrm{recall}}$$ (6)

Exact conditional logistic regression (PROC LOGISTIC) was used to examine the effects of *PRNP* genotype and stage of preclinical infection on the probability of true positive detection by the standardized RT-QuIC protocol and optimum diagnostic call criteria as determined for each substrate. The model included the class variables genotype at codon 96 (GG96 vs. xS96) and stage of preclinical infection (early vs. late) as well as the interaction term; Generalized Linear Model (GLM) parameterization was used. Strata were defined by substrate source (MNPROtein and NWHC). The overall model was found to be highly significant as determined by exact methods (Joint Score statistic = 30.0334, *P*_*exact*_ < 0.0001). Subsequent comparisons of interest were made by least squares means estimation (LSMESTIMATE option; degrees freedom = 3, Joint Chi-Square = 21.85, *P* < 0.0001) with multiple comparison adjustments using the Holm method (i.e., stepdown Bonferroni). Significant differences from multiple comparisons were defined as *P*_*Holm*_ < 0.05 and effect size expressed as odds ratios (OR) with 95% lower and upper confidence limits given in brackets [LCL, UCL].

## Results and discussion

### Pre-analytic sample preparation

Amplification of prion seeds in RT-QuIC reactions can be inhibited by tissue components when sample material is too concentrated. To minimize this type of inhibition, RT-QuIC reactions are frequently seeded using a 0.1% (10^−3^ dilution) tissue homogenate [16,27]. While this practice may be necessary to overcome inhibition of the reaction by tissue components, it also reduces the overall amount of tissue that is tested. To account for possible differences associated with the archival nature of the samples used in this study, RT-QuIC reactions were seeded with serial dilutions of rectal mucosa from one of the archived late-stage preclinical CWD infected deer. As seen in Fig 1A (green box plots), no seeding reactions were observed at the 10^−1^ dilution but were observed in all wells at sample dilutions of 10^−2^ and 10^−3^. Reaction lag times for the 10^−2^ dilution were, however, longer than those observed for the 10^−3^ dilution. Thus, in general agreement with previous studies, inhibition by tissue components was still evident in this sample as a 1% homogenate.

Differential protein precipitation using sodium phosphotungstic acid (NaPTA) has been shown to enrich misfolded PrP from surrounding tissue components and yield improved results for both immunoblot [28] and RT-QuIC [20]. When applied to the same sample dilutions above, reactions were observed in all wells seeded with sample at the 10^−1^ dilution with the shortest lag times observed for the 10^−2^ dilution (Fig 1A, blue box plots).

A concern was that this improvement in detection could be limited to samples with a high prion titer, where relatively high amounts of PrP^CWD^ may compensate for losses associated with additional processing. To address this, the 10% rectal mucosa homogenate of the CWD-positive deer was diluted into the 10% homogenate of a CWD-ND deer to maintain tissue context. Thus, three mock samples were created that contained PrP^CWD^ at levels 100 times, 1,000 times, and 10,000 times more dilute than the original sample. Each mock sample was then tested in parallel (Fig 1B) to compare detection limits of the dilution in buffer method (10^−3^ dilution, green box plots) and the resuspended NaPTA precipitation method (reactions seeded with the equivalent of 10^−2^ dilution, blue box plots). The resuspended NaPTA precipitation method detected seeding activity an order of magnitude more dilute than could be detected by dilution in buffer alone (Fig 1B). As such, the resuspended NaPTA precipitation method was added as a pre-analytic step.

### Application of the standardized RT-QuIC protocol

The same homogenate preparation of the mock biopsy sample from each deer was tested in two independent assays, each using a different source of Ha90 rPrP substrate: the commercially sourced MNPROtein substrate and the laboratory produced NWHC substrate. The purity, quality, and proper handling of the rPrP substrate is a major factor and potential source of variability in RT-QuIC assay results. By testing each sample with more than one source of substrate, each acquired through commercial shipping on dry ice, we were able to evaluate the reproducibility of the assay results as well as more closely examine how reaction parameters and outcomes may be affected by different sources of substrate.

#### Diagnostic criteria for the RT-QuIC assay

The aggregation reaction of Ha90 rPrP is realized as an exponential increase in ThT fluorescence. A ‘positive’ diagnostic call should distinguish reactions occurring in response to PrP^CWD^ seeds present in the homogenate sample (seeded reactions) from those that variably occur in the apparent absence of PrP^CWD^ seeds (spontaneous reactions), especially at prolonged assay run times.

**Fig 2:**
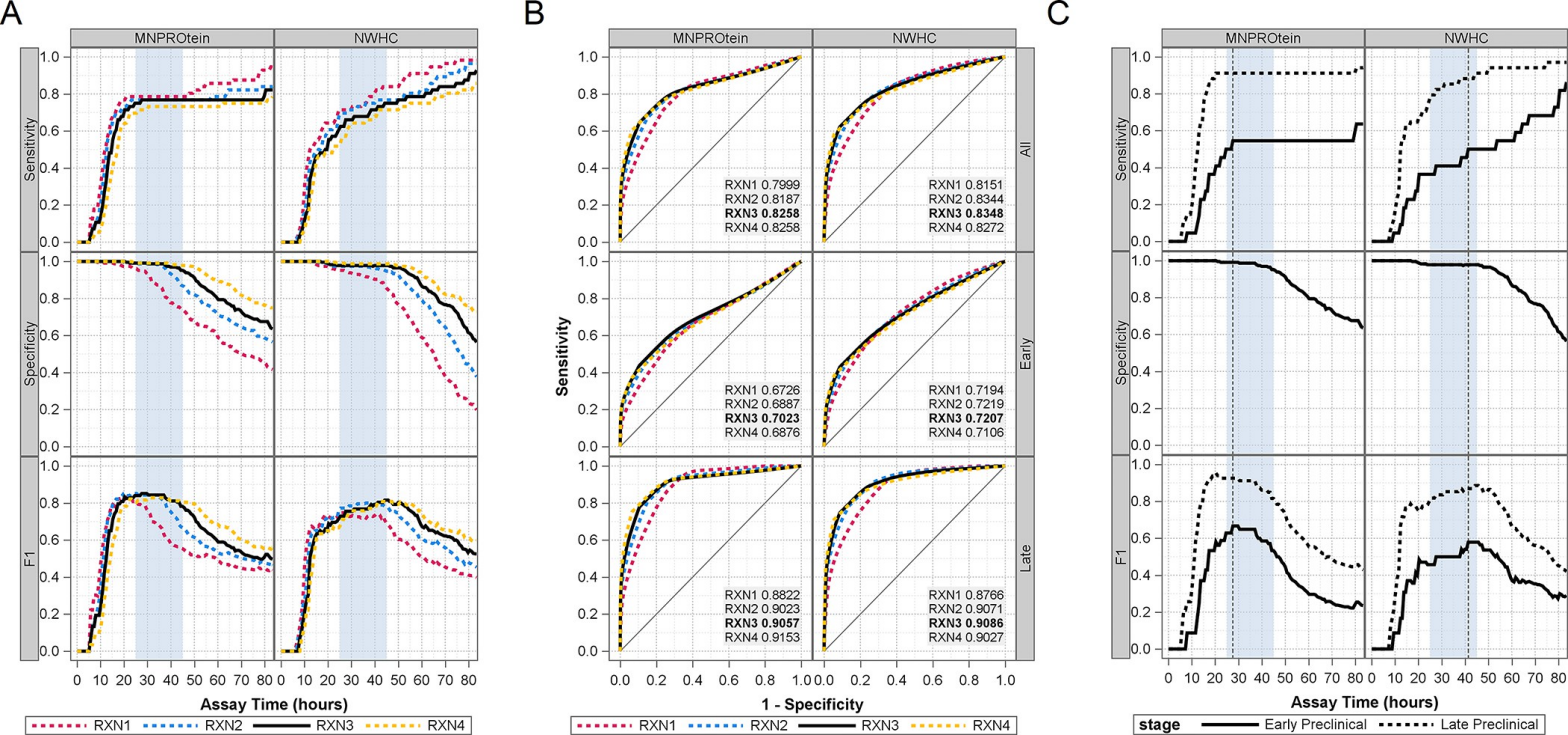
Effects of assay reaction criterion choices on diagnostic performance measures. (A) Sensitivity, Specificity, and F1 curves in detecting chronic wasting disease in preclinical white-tailed deer (*Odocoileus virginianus*) were determined over time for each choice of real-time quaking-induced conversion (RT-QuIC) reaction (RXN) criterion. The RXN criterions for calling a sample’s results ‘positive’ were defined as the detection of substrate aggregation in at least one (RXN1), two (RXN2), three (RXN3), or all four (RXN4) replicate wells. The range of assay hours producing near-optimal measures of diagnostic performance is highlighted (vertical light blue shading). (B) The receiver operator characteristic (ROC) curves for all preclinical, early preclinical, and late preclinical stages of infection as determined from each choice of RXN criterion. The calculated area under the curve (AUC) for each RXN criterion is provided as insets; the optimal choice of RXN3 is bolded. (C) Sensitivity, Specificity, and F1 curves over time for RT-QuIC diagnostic calls based on the RXN3 criterion separated for early or late preclinical cases. The earliest assay time that produced the maximum F1 value for early preclinical deer (vertical dotted gray lines) occurred at 27.55 hours when using the MNPROtein substrate and 41.325 hours when using the NWHC substrate.

**Fig 3:**
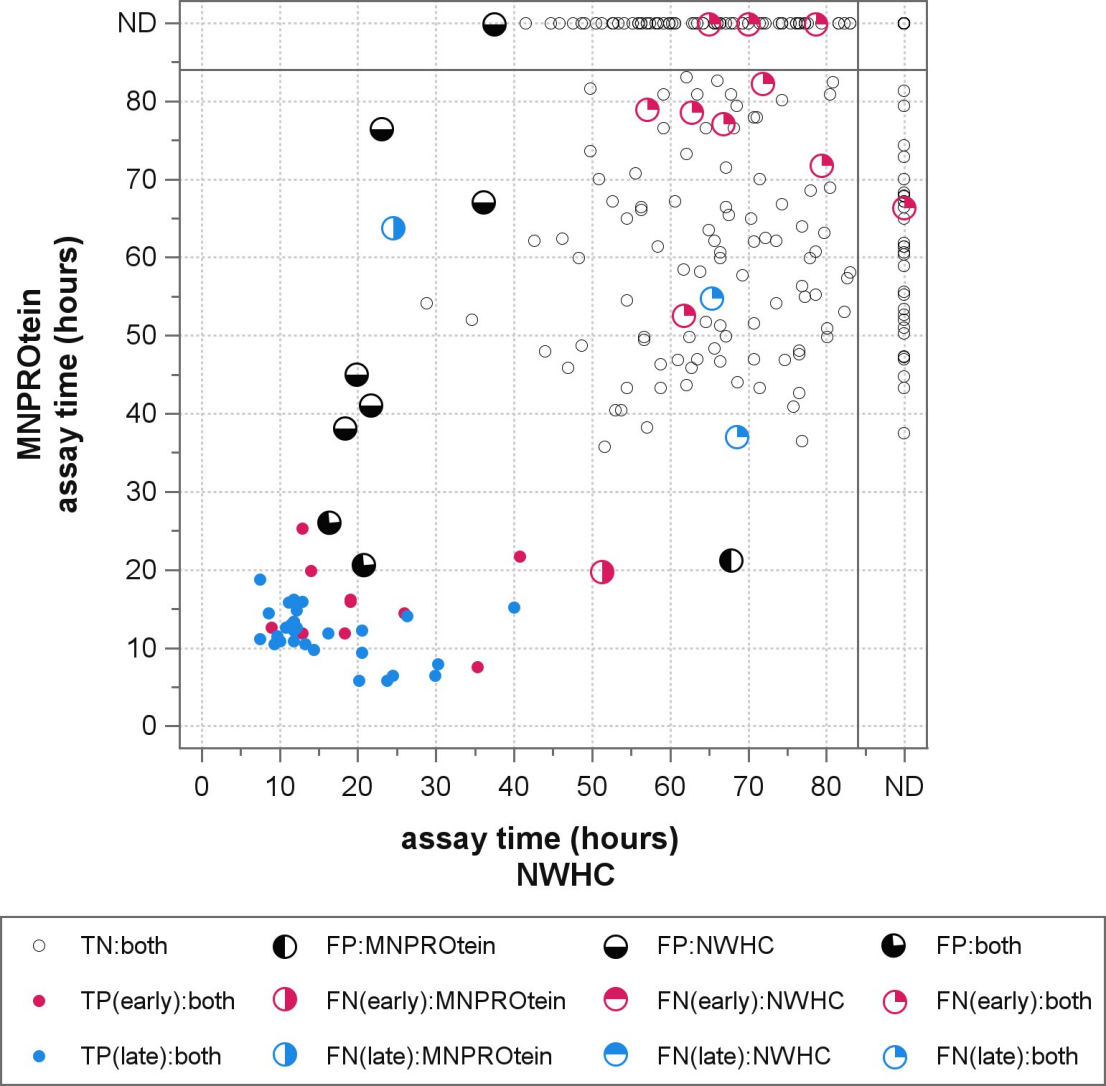
Relationship of paired RT-QuIC median lag times to WTD infection status. Median RT-QuIC lag times from each rectal mucosal sample are plotted by substrate (MNPROtein vs. NWHC), using color to indicate the deer’s stage of preclinical infection (early in red; late in blue). Samples in which an RT-QuIC reaction was not observed in any of the four replicate wells are indicated on the corresponding substrate axis as ND; for 21 samples, no reactions were detected in either substrate. The shading scheme defined in the legend indicates the concordance of the substrate-optimized RT-QuIC diagnostic call (minimum three reactions detected by 27.55 hours when using MNPROtein and by 41.325 hours when using NWHC) with the deer’s infection stage. Note that the paired median lag times from samples producing TP diagnostic calls generally cluster in the bottom left quadrant of the graph (early assay run times) in contrast to those from TN diagnostic calls in the upper right (late assay run times). Consistency between paired median lag times and deer infection status is expected when PrP^CWD^ seed is genuinely present or absent in the tested rectal mucosal sample. Note, too, that the paired median lag times for 12 of 14 FN diagnostic calls were clustered with the paired median lag times of TN calls, possibly indicating the absence of PrP^CWD^ seed in these samples. Likewise, two of nine FP diagnostic calls are closest to samples producing TP diagnostic calls, possibly indicating the presence of PrP^CWD^ seeds in these samples.

RT-QuIC assays are generally run using multiple replicate reactions for each sample, which helps distinguish seeded from spontaneous reactions. In this study, each sample was tested in quadruplicate reactions with ThT fluorescence measurements taken at regular intervals over an assay duration of ~83 hours. For each sample at each timepoint, the number of replicate wells exceeding *ThT*_*crit*_ was recorded. Diagnostic calls for each sample were then made at each assay time based on each of four possible criterions of ‘reactivity,’ where RXN# represents the minimum number of reacting wells required to call a positive diagnosis. Graphs of all quadruplicate reactions (S1 Figs 001–026) and the associated reaction data are provided at the Ag Data Commons repository (DOI: 10.15482/USDA.ADC/24727866). The resulting RT-QuIC diagnostic calls were compared to the gold-standard diagnoses to calculate the sensitivity, specificity, and F1 score for each reactivity criterion over time (Fig 2A). As an objective method to compare reactivity criterions (RXN#), receiver operating characteristic (ROC) plots were generated for each, and area under the curve (AUC) was calculated. For each substrate source, a minimum reactivity criterion requiring 3 of 4 replicates to be above threshold for a positive call (RXN3) yielded the highest AUC (Fig 2B, top row), an indication of best agreement with the gold standard results. This is visually apparent also in Fig 2A, where the criterions requiring only 1 or 2 positive replicates resulted in early decreases in specificity and the criterion requiring all 4 replicates to be positive prolonged the time necessary to maximize sensitivity with only minor improvement of specificity.

When choosing an assay criterion, it is also important to consider the impact that stage of infection may have on diagnostic performance. It is an observed property of the RT-QuIC assay that slower reaction lag times and inconsistent replicate reactions are produced when known prion-positive samples are serially diluted; this is assumed to predict the reaction properties of the minimally prion-positive natural sample. For this reason, these performance metrics were also calculated controlling for preclinical infection stage (Fig 2B, rows 2,3). For both substrate sources, the RXN3 criterion either produced the maximum AUC, produced an AUC which was not statistically distinguishable from the maximum (Fig 2B, Early Preclinical, NWHC: RXN3-RXN2, Chi-Square = 0.1417, *P* = 0.7066), and in one instance produced an AUC that was less than that produced by RXN4 (Fig 2B, Late Preclinical, MNPROtein: RXN3-RXN4, Chi-Square = 88.8611, *P*<0.0001).

Used as a diagnostic, it is also essential to establish an assay cutoff time so that aggregation events are only evaluated at assay durations where seeded and spontaneous reactions are reliably differentiated. There are many diagnostic performance criteria that have been used to objectively estimate the optimal assay cut point. As one example, we identified the cut point as the earliest timepoint at which the F1 score was maximized for early preclinical animals based on the RXN3 reactivity criterion. This resulted in cutoff times of 27.55 h for the MNPROtein substrate and 41.325 h for the NWHC substrate; depicted with vertical lines on Fig 2C. The decision to determine cutoff times using early preclinical cases was made because these cases are both more difficult to detect and show more impactful changes in response to assay cutoff time (Fig 2C). Performance metrics for the overall and late preclinical sample sets were not substantially reduced from their respective maxima at the timepoints identified in the early preclinical set (Fig 2A and 2C) and both substrates were characterized by a “window” of near-optimal assay performance between 25 and 45 hours. This window of time is most clearly visualized as a plateau in the F1 score and is highlighted with blue shading (Fig 2A and 2C). Despite this, the differences apparent in early preclinical cases demonstrate the importance of specific consideration of this infection stage during optimization of the assay cutoff time.

#### Diagnostic performance

Using the RXN3 reactivity criterion and the cutoff times selected above, the performance metrics measured for the standardized RT-QuIC protocol using samples of rectal mucosa from farmed WTD produced an overall preclinical diagnostic sensitivity of 77% and specificity of 99% with the MNPROtein substrate; and 73% and 98% with the NWHC substrate, respectively. The sensitivity, specificity, and 95% confidence intervals for early, late, and overall preclinical stages of infection are provided in the S1 Table at 10-hour intervals over the duration of the assay.

Preclinical infection stage was a significant predictor of assay sensitivity (Score statistic = 7.5296, *P*_*exact*_ = 0.0108), with detection sensitivities of the respective substrate sources of 91% and 88% for late preclinical deer, and 55% and 50% for early preclinical deer (Table 2). This held true for deer bearing *PRNP* alleles wild type for codon 96 (GG96) as well as for deer carrying at least one S96 allele (xS96) (early vs late, OR: xS96 = 0.089 [0.013, 0.591], GG96 = 0.205 [0.061, 0.680], each: *P*_*Holm*_ = 0.0386). However, the effect of *PRNP* genotype on assay performance segregated by infection stage of the animals tested.

**Table 2:** Substrate-optimized diagnostic performance of the standardized RT-QuIC protocol for white-tailed deer rectal mucosa.

Performance measure	Preclinical stage	*PRNP* codon 96	Total	MNRPOtein			NWHC		
				Estimate	Lower	Upper	Estimate	Lower	Upper
**Sensitivity**	**All**	*overall*	**56**	**0.7679**	0.6358	0.8702	**0.7321**	0.5970	0.8417
		GG	40	**0.8750**	0.7320	0.9581	**0.8000**	0.6435	0.9095
		GS	10	**0.5000**	0.1871	0.8129	**0.5000**	0.1871	0.8129
		SS	3	**0.3333**	0.0084	0.9057	**0.3333**	0.0084	0.9057
		xS	13	**0.4615**	0.1922	0.7487	**0.4615**	0.1922	0.7487
		*unk*	3	**0.6667**	0.0943	0.9916	**1.0000**	0.2924	1.0000
	**Early**	*overall*	**22**	**0.5455**	0.3221	0.7561	**0.5000**	0.2822	0.7178
		GG	14	**0.7143**	0.4190	0.9161	**0.6429**	0.3514	0.8724
		GS	5	**0.2000**	0.0051	0.7164	**0.2000**	0.0051	0.7164
		SS	3	**0.3333**	0.0084	0.9057	**0.3333**	0.0084	0.9057
		xS	8	**0.2500**	0.0319	0.6509	**0.2500**	0.0319	0.6509
		*unk*	0	***ne***	* *	* *	***ne***		
	**Late**	*overall*	**34**	**0.9118**	0.7632	0.9814	**0.8824**	0.7255	0.9670
		GG	26	**0.9615**	0.8036	0.9990	**0.8846**	0.6985	0.9755
		GS	5	**0.8000**	0.2836	0.9949	**0.8000**	0.2836	0.9949
		SS	0	***ne***			***ne***		
		xS	5	**0.8000**	0.2836	0.9949	**0.8000**	0.2836	0.9949
		*unk*	3	**0.6667**	0.0943	0.9916	**1.0000**	0.2924	1.0000
**Specificity**	**All**	*overall*	**228**	**0.9912**	0.9687	0.9989	**0.9781**	0.9496	0.9928
		GG	48	**0.9792**	0.8893	0.9995	**1.0000**	0.9260	1.0000
		GS	27	**0.9630**	0.8103	0.9991	**0.8889**	0.7084	0.9765
		SS	4	**1.0000**	0.3976	1.0000	**1.0000**	0.3976	1.0000
		xS	31	**0.9677**	0.8330	0.9992	**0.9032**	0.7425	0.9796
		*unk*	149	**1.0000**	0.9755	1.0000	**0.9866**	0.9524	0.9984
	**Early**	*overall*	**228**	**0.9912**	0.9687	0.9989	**0.9781**	0.9496	0.9928
		GG	48	**0.9792**	0.8893	0.9995	**1.0000**	0.9260	1.0000
		GS	27	**0.9630**	0.8103	0.9991	**0.8889**	0.7084	0.9765
		SS	4	**1.0000**	0.3976	1.0000	**1.0000**	0.3976	1.0000
		xS	31	**0.9677**	0.8330	0.9992	**0.9032**	0.7425	0.9796
		*unk*	149	**1.0000**	0.9755	1.0000	**0.9866**	0.9524	0.9984
	**Late**	*overall*	**228**	**0.9912**	0.9687	0.9989	**0.9781**	0.9496	0.9928
		GG	48	**0.9792**	0.8893	0.9995	**1.0000**	0.9260	1.0000
		GS	27	**0.9630**	0.8103	0.9991	**0.8889**	0.7084	0.9765
		SS	4	**1.0000**	0.3976	1.0000	**1.0000**	0.3976	1.0000
		xS	31	**0.9677**	0.8330	0.9992	**0.9032**	0.7425	0.9796
		*unk*	149	**1.0000**	0.9755	1.0000	**0.9866**	0.9524	0.9984

Estimates of the sensitivity and specificity are reported here as binomial proportions (bold text) with exact 95% confidence limits. The data are grouped by source of the substrate (MNPROtein, NWHC) and stratified by stage of preclinical infection and *PRNP* genotype at codon 96 (G = glycine; S = serine; x = G or S). Colored data bars (scaled 0 to 1; sensitivity = blue, specificity = yellow) are provided to aid visualization over these strata. Calculations were based on substrate-specific optimized diagnostic criterions where reactions were detected in a minimum of three of four sample replicates at 27.55 hours when using the MNPROTein substrate and at 41.325 hours when using the NWHC substrate. Gold standard results were based on the detection of PrP^CWD^ in the MRPLN and obex tissues by IHC. *PRNP* genotypes were not available (*unk*) for three deer diagnosed with CWD by IHC and 149 deer in which CWD was not detected by IHC. Sensitivity was not estimated (*ne*) for rows with zero deer.

At the late preclinical stage of infection, 96% and 88% of GG96 deer in the respective substrates and 4 of 5 GS96 deer (80%, both substrates) were detected by the RT-QuIC assay. Given the small number of xS96 deer in the archival sample set (Table 2), it is not possible to determine if the xS96 genotype has any significant impact on detection of late preclinical infection from biopsies of the rectal mucosa (late, xS96 vs GG96, OR = 0.382 [0.064, 2.280], *P*_*Holm*_ = 0.2913). However, significant differences were apparent between genotypes in early preclinical animals (early, xS96 vs GG96, OR = 0.166 [0.042, 0.648], *P*_*Holm*_ = 0.0386), where GG96 deer were detected with sensitivities of 71% and 64% in the respective substrates, but only 2 of 8 xS96 deer were detected (25%, both substrates).

Three animals bearing the QH genotype at codon 95 were present in this study group, one of which was diagnosed as early preclinical by CWD-IHC. This animal also carried the GS96 polymorphism and was not detected by RT-QuIC (Table 1).

### Limitations and test assay-vs-gold standard discrepancies

#### PrP^CWD^ seeds in rectal mucosa from deer with early preclinical infection: Not detected or not present?

Previous research has demonstrated that the MRPLN is one of the first tissues to accumulate prions following infection [4,5]. For this reason, this tissue is favored for the detection of early-stage prion infection and, along with the obex, is used for official CWD diagnoses [9]. It is reasonable to expect that a portion of early preclinical infections in the current study may not yet have accumulated prions in the rectal mucosal at the time of depopulation. This is supported by a high proportion of false negative RT-QuIC diagnostic calls in early preclinical cases (Fig 3).

Similar trends have been observed in prior studies assessing CWD-IHC of rectal mucosal biopsies as a potential diagnostic method in deer. The IHC-based definitions of early and late preclinical infections were identical to those outlined for the current study. A 2009 study was able to detect PrP^CWD^ in 92% (45/49) of late preclinical deer, but only 14% (1/7) of early preclinical deer [15]. In a 2012 meta-analysis of four WTD depopulations, PrP^CWD^ was detected in RAMALT in 91% (71/78) of late preclinical deer but only 36% (21/58) of early preclinical deer [12]. A similar difference was observed in a 2016 study where late preclinical deer were detected with a sensitivity of 58% (122/209), which was reduced to only 8% (7/81) in early preclinical deer [14]. Analogous results have also been observed in several CWD-IHC studies of rectal mucosa from elk, though these studies had smaller proportions of early preclinical infections, suggesting possible differences in prion accumulation pattern and time course for elk [16,19,29]. These studies demonstrate that PrP^CWD^ accumulation in RAMALT generally lags accumulation in MRPLN. For this reason, detection of CWD in early preclinical animals via antemortem-accessible tissues, such as RAMALT, is likely to be inherently less sensitive than assay of more optimal, postmortem-only tissues.

A second commonality apparent in these studies is the effect of *PRNP* genotype on assay sensitivity. Several studies have shown that the S96 allele results in a prolonged course of infection with delayed and reduced accumulation of PrP^CWD^ in most tissues [13,30,31]. Perhaps the most concise example is found in an oral inoculation study of WTD with differing codon 96 polymorphisms [13]. At the first assay timepoint of 253 days post inoculation (dpi), 8 of 10 GG96 deer had become CWD-IHC-positive in the RAMALT, while PrP^CWD^ was detected in the RAMALT of only 1 of 8 GS96 deer. A little over 7 months later, at 477 dpi, PrP^CWD^ was detected in the RAMALT of 6 of the 8 GS96 deer tested by CWD-IHC. By the conclusion of the study, at 751 dpi, PrP^CWD^ had been detected in the RAMALT of 9 of 10 GG96 deer and 7 of 8 GS96 deer. The study included only three inoculated SS96 deer, for which no PrP^CWD^ accumulation in the RAMALT was observed during the study [13]. Reduced CWD-IHC sensitivity for rectal mucosal samples from deer carrying at least one S96 allele was also observed in each of the screening studies discussed above [12,14,15].

It can be difficult to extricate the respective influences of genotype and preclinical infection stage on assay sensitivity, as those animals bearing polymorphisms associated with delayed distribution kinetics are the same animals most likely to be less progressed in infection at any given time. In a study by Keane et al. [15], all seven early preclinical infections bore the GS96 genotype, while all CWD-positive GG96 deer were late preclinical infections. Interestingly, 5 of the 7 early preclinical GS96 deer were detected by CWD-IHC of the rectal mucosa. In another study by Thomsen et al. [12], the overall preclinical diagnostic sensitivity of GS96 deer was 42% (8/19), which segregated by infection stage to be 71% (5/7) for late preclinical deer but only 23% (3/13) for early preclinical deer. Though limited by smaller sample sizes, these data imply that the predominant impact of the S96 allele on CWD diagnosis using samples of the rectal mucosa is realized through delayed progression and reduced accumulation of PrP^CWD^ rather than incompatibility of S96 PrP^CWD^ with detection by IHC.

The RT-QuIC assay has been explored as a means of addressing the limited diagnostic sensitivity of conventional immunoassays including CWD-IHC. In a laboratory setting, the limit of detection by RT-QuIC has been shown to be lower than immunoassay, and comparable to mouse bioassay [32,33]. Animal screening studies of RT-QuIC specifically using rectal mucosa have also demonstrated improved sensitivity when tested in parallel with CWD-IHC. One study using rectal mucosal samples from elk reported the sensitivity of CWD-IHC to be 52% which was improved to 76% by RT-QuIC [22]. A similar study in deer reported an increased correlation with CWD positivity of 0.55 for RT-QuIC from 0.38 for IHC [14]. Finally, in a study using biopsy samples of rectal mucosa collected longitudinally after oral inoculation, infection was detected by RT-QuIC before CWD-IHC in 74% of deer, with the remainder detected concurrently by both methods [20]. This study also demonstrated that pre-analytic preparation of samples as resuspended NaPTA precipitate improved the sensitivity of RT-QuIC [20].

In the current study, pre-analytic preparation of samples as resuspended NaPTA precipitate yielded overall sensitivities (77% and 73% for respective substrates) similar to or better than previous studies using CWD-IHC (80% [15], 68% [12], and 54% [14]). Our results suggest an advantage for RT-QuIC in early preclinical cases, achieving 50–55% sensitivity compared to the 8–36% reported from CWD-IHC studies [12,14,15]. However, early preclinical deer bearing xS96 alleles still present a challenge for detecting CWD infections by rectal mucosal biopsy (25% sensitivity). While nominally an improvement over the 14% and 23% reported for IHC screening of analogous samples, the sample sizes in this specific subset were too small to provide significance. Nonetheless, detection of the earliest cases using samples of rectal mucosa, especially in xS96 deer, remains difficult irrespective of the assay method used.

A recent study utilizing samples of rectal mucosa from WTD produced a diagnostic sensitivity of 86% (18/21) for RT-QuIC assay [21]. Though the number of deer in each infection stage and *PRNP* genotype subgroup was small, the results also align with improved detection in xS96 and/or early preclinical deer. Of note, the initial homogenates made in that study used 200 mg of rectal mucosa as compared to ~100 mg used in this study. It is possible that initial homogenates made using larger amounts of a given sample may improve the likelihood of including PrP^CWD^ seeds in early cases where its presence may be particularly sparse and unevenly distributed. Spatial distribution has been demonstrated to impact prion detection in tonsil tissue, where prions were detected in 87% of animals when whole tonsil samples were examined compared to only 72% of animals for which only biopsies were tested, with the effect more pronounced in early-preclinical animals at 83% for whole tonsil and 55% for biopsy only [11]. These data suggest that preparing the initial homogenate using a larger sample size may increase RT-QuIC assay sensitivity where feasible.

#### Could false positives be truly positive in rectal mucosa?

A limitation in the current study is that all samples were collected from deer on farms positive for CWD prior to depopulation. All prion diseases exhibit an initial period of incubation during which infection can be undetectable. It is probable that a portion of the deer in this study included infected deer in which PrP^CWD^ was not detected in the MRPLNs and obex by IHC. It is also known that amplification assays, such as RT-QuIC, are capable of detecting PrP^CWD^ seeds below the threshold for detecting PrP^CWD^ via immunoassay in a given sample [32].

In this study, aggregation reactions were observed in several samples of rectal mucosa from deer in which PrP^CWD^ had not been detected in MRPLNs or obex by IHC. In fact, two of these samples displayed highly reproducible reactivity at assay timepoints well below the calculated optimal assay cutoff times in both substrates. As highlighted in Fig 3, the paired median lag times for these two samples (3/4 filled black circles) cluster closely with those from known infected animals (filled red and blue circles). This suggests that these sample homogenates in fact harbor genuine PrP^CWD^ seeds. To confirm this, the remainders of these homogenates are currently being tested for infectivity by intracerebral inoculation of cervidized transgenic mice. Should these samples contain CWD prions, the performance metrics for this standardized RT-QuIC protocol could be updated and would result in improved specificity.

Other aggregation reactions resulting in false positive diagnostic calls (Fig 3, half-filled black circles) were less consistent between substrates and had longer, more variable lag times (Fig 3). A previous study using RT-QuIC and rectal mucosa samples from 39 deer from a CWD-naïve herd found 0 false positives in a 24 hour assay but an apparent false positive rate of 6.1% for samples from deer originating from a herd quarantined due to CWD [14]. This discrepancy suggests that some prolonged lag time reactions may not necessarily be spontaneous in origin. Expanding such experimental methodologies which include testing of naïve herds to include larger sample sets and longer assay run times would provide valuable information to differentiate genuinely seeded late reactions from spontaneous reactions in samples from early preclinical infections containing minimal PrP^CWD^ seeds.

## Conclusions

The preclinical diagnostic performance metrics observed for this standardized RT-QuIC protocol broadly agree with multiple previous studies and produced improved sensitivity compared to CWD-IHC under comparable conditions but in less than 30 hours and in a 96-well format. This sensitivity improvement is not yet able to fully compensate for the inherently delayed prion accumulation in the RAMALT relative to the MRPLN. While assay of the MRPLN may remain the more sensitive test for the detection of prion disease in a single animal at a single timepoint, there are management scenarios where diagnosis by rectal mucosa biopsy would be advantageous. The fact that RT-QuIC using rectal mucosa samples is a repeatable antemortem assay allows for the application of screening and monitoring strategies that are simply not possible with a single-timepoint postmortem assay.

## Supporting information

S1 Table Time course of substrate-optimized diagnostic performance of the standardized RT-QuIC protocol for white-tailed deer rectal mucosa. Estimates of the sensitivity and specificity are reported here as binomial proportions (bold text) with exact 95% confidence limits. Colored data bars are also provided to aid visualization over time (scaled 0 to 1; sensitivity = blue, specificity = yellow). The data are grouped by substrate source (MNPROtein, NWHC) and stratified by stage of preclinical infection. Calculations were based on contingency tables where a positive result from the RT-QuIC assay was defined as detection of a minimum of three (of four) replicate reactions per sample. Gold standard results were based on the detection of PrP^CWD^ in the MRPLN and obex tissues by IHC. The boxed-out time for each substrate was the time that maximized the F1 score for detecting cases of early preclinical infection.
(XLSX)
